# Synergistic effects of recombinant expressed Fowlicidin and Thymosin α1 hybrid peptides in modulating inflammation and infection in avian macrophages

**DOI:** 10.3389/fmicb.2025.1568451

**Published:** 2025-04-16

**Authors:** Baseer Ahmad, Zaheer Abbas, Wei Xubiao, Kashif Hussain, Atif Rehman, Si Dayong, Asghar Abbas, Christina Hölzel, Zhang Rijun

**Affiliations:** ^1^Laboratory of Feed Biotechnology, State Key Laboratory of Animal Nutrition and Feeding, College of Animal Science and Technology, China Agricultural University, Beijing, China; ^2^Faculty of Animal and Veterinary Sciences, Muhammad Nawaz Shareef University of Agriculture, Multan, Pakistan; ^3^Institute for Animal Breeding and Animal Husbandry, Kiel University, Kiel, Germany

**Keywords:** hybrid peptide, yeast expression, anti-inflammatory, antimicrobial, endotoxin neutralization

## Abstract

The hybrid FowlTα1 peptide represents a promising biomolecule synthesized from two naturally occurring peptides, namely Fowlicidins (Fowl) and Thymosin α1 (Tα1). This particular peptide exhibits remarkable anti-inflammatory and antimicrobial properties and demonstrates the capacity to effectively interact with lipopolysaccharide (LPS), while simultaneously inducing minimal cytotoxicity and hemolytic repercussions. Despite its potential, the high cost of this peptide has limited its use. To overcome this limitation, the present study developed a cost-effective and biocompatible method for expressing the FowlTα1 peptide in *Pichia pastoris* (*P. pastoris*). We obtained a transgenic strain of the hybrid FowlTα1 peptide with a predicted molecular weight of 3.1 kDa. The FowlTα1 peptide was purified followed by reverse-phase high-performance liquid chromatography (RP-HPLC), yielding 7.2 mg with a purity of 98.2%. Furthermore, physiochemical and structural analysis revealed an amphipathic helical configuration that enhances bioactivity. Moreover, in LPS-stimulated HD11 macrophages, the hybrid FowlTα1 peptide significantly reduced the release of nitric oxide (NO), TNF-α, IL-6, and IL-1β in a dose-dependent manner (*p < 0.05*) and displayed robust antimicrobial activity against *Escherichia coli* (*E. coli*) compared to conventional antibiotic. Overall, the results of this study highlighted the production method and potential of the FowlTα1 peptide as a novel therapeutic agent for antimicrobial, anti-inflammatory, and anti-endotoxin applications.

## Introduction

Antibiotics are extensively utilized as a feed additive to promote growth and veterinary therapy by livestock worldwide. In certain countries, antibiotic use in livestock is expected to exceed 50% of the total national consumption, contributing to a global estimate of 63,151 tons (Klein et al., 2024). As a result, occurrence of antibiotic residues in food and the emergence of infections caused by antimicrobial-resistant or multidrug-resistant (MDR) have become significant challenges (Treiber and Beranek-Knauer, 2021). Lipopolysaccharide (LPS), also known as endotoxin, is an integral component of the outer membrane of Gram-negative bacteria. It is released upon the destruction of the bacteria and can trigger various adverse conditions. These include the secretion of pro-inflammatory cytokines through immune response activation, sickness, reduced weight gain, decreased feed intake, altered animal behavior (Sun and Shang, 2015) as well as severe outcomes such as lethal shock, leukopenia, and sepsis (Callaway et al., 2021; Candelli et al., 2021). However, it is important to note that systemic effects typically occur only after parenteral exposure. LPS is also known to influence the gut microbiome (Raetz and Whitfield, 2002; Wei et al., 2023; Huang et al., 2024; Li et al., 2025) and some of its effects, such as reduced weight gain and feed intake, may be partially mediated through interactions with the microbiome. Consequently, there is a compelling need to explore and develop viable alternatives to antibiotics that possess both antibacterial and LPS-neutralizing properties for use as feed additives in livestock (Callaway et al., 2021; Stoica and Cox, 2021). The utilization of antimicrobial peptides (AMPs) such as cathelicidins and defensins is a promising strategy. These peptides demonstrate the capability to effectively eliminate a broad spectrum of microorganisms, including MDR bacteria, yeast, fungi, protozoa and viruses (Girdhar et al., 2024). Compared with conventional antibiotics, AMPs implement microbial eradication by multiple mechanisms targeting both the cell membrane and intracellular components. Extraordinarily, despite millions of years of co-evolution most microorganisms remain highly susceptible to AMP-mediated elimination (Lewies et al., 2019). Among the AMPs, Fowl a cathelicidin expressed in the chicken intestine, is well-known for its antimicrobial and anti-inflammatory properties (Nazeer et al., 2021). The Fowl peptide assumes a pivotal role as a primary line against local and systemic microbial infections. Notably, the Fowl peptide binds LPS or endotoxin, neutralizing its biological activity by suppressing interleukin-6 (IL-6), interleukin-1 beta (IL-1β) and LPS-induced tumor necrosis factor (TNF-α) (Sarandeses et al., 2003; Xiao et al., 2009). Consequently, the Fowl peptide demonstrates significant potential as a highly favourable peptide for controlling inflammation and mitigating the impact of endotoxin. Thymosin α1 (Tα1) is another peptide derived from the natural thymic peptide comprising 28 amino acids (Garaci et al., 2007). The Tα1 peptide is widely recognized for its critical role in modulating infectious diseases, functioning as an immune response regulator and exerting a primary influence on cells of the innate immune system (Saugar et al., 2006; Romani et al., 2007).

Hybridizing active parts of dissimilar AMPs is the most effective method to obtain hybrid peptides with elevated antibacterial, anti-inflammatory, and less cytotoxic abilities (Wei et al., 2018; Erdem Büyükkiraz and Kesmen, 2022). There are three distinct pathways exist for the isolation of AMPs; (i) extraction from naturally available assets (ii) chemical synthesis (iii) production by DNA recombinant technology (Parachin et al., 2012; Ahmad et al., 2014). In comparison to bacterial expression systems, methylotrophic yeast (*P. pastoris*) has gained widespread use for the expression of AMPs due to its high yield and cost-effective production (Gellissen et al., 2005). Furthermore, this yeast exhibits rapid growth in economical media, achieves high cell densities, and is amenable to genetic modification. As a eukaryotic system, it also facilitates post-translational modifications, such as protein folding, disulfide bridge formation, and glycosylation (Ahmad et al., 2019).

Previously, our laboratory designed and expressed hybrid peptides, including LL-37Tα1, CATH-2TP5, DEFB-TP5 (Ahmad et al., 2019; Ahmad et al., 2020a; Ahmad et al., 2020b) and CLTP (Cheng et al., 2023) successfully in yeast expression systems. The anti-inflammatory activities of these peptides were subsequently evaluated using both in vitro and in vivo models (Roslansky and Novitsky, 1991). Consequently, in the present study, we hypothesized that the combination of Fowl (19 amino acids) and Tα1 (7 amino acids) may have augmented its ability for LPS neutralization and its immunomodulatory, anti-inflammatory activity, and may have lowered its cytotoxic effects. Hence, we synthesized and expressed the hybrid peptide FowlTα1 in a methylotrophic yeast expression system and investigated its bioactivities.

## Materials and methods

### Materials

#### Strains, vectors, and reagents

The expression hosts *P. pastoris* (Strain *SMD 1168*), *Escherichia coli* (*E. coli* DH5α), the methanol-induceable cloning plasmid pPICZαA that includes a Zeocin-resistance gene allowing selection, and the antibiotic Zeocin was purchased from Invitrogen, Carlsbad, CA, USA. The restriction enzymes *EcoR I, Not I, Sac I* (TaKaRa Biotechnology, Dalian, China) and PCR reagents, DNA Marker (50 and 1,000 bp) were obtained from Tiangen Biotech (Beijing, China). The *E. coli* endotoxin (O55: B5) was provided by Sigma, USA. The Gel Extraction Kit, Plasmid Mini Kit, Yeast DNA Extraction Kit, and Protein Markers were purchased from Sangon Biotech, Shanghai, China.

#### Design and sequence analysis of hybrid peptide FowlTα1

The hybrid peptides were designed by combining the active centers of Fowl and Tα1. The physio-chemical properties were calculated online using ProParam (ExPASy Proteomics Server^1^); pepdraw.com. The helical wheel projection and toxicity evaluation for the sequence of parental and hybrid peptides were obtained using https://heliquest.ipmc.cnrs.fr/index.html and https://webs.iiitd.edu.in/raghava/toxinpred/algo.php, respectively.

#### Assembly of hybrid recombinant expression plasmid pPICZαA-FowlTα1

The amino acid sequence of hybrid peptide FowlTα1 and the preferred codon of *P. pastoris* were optimized using JAVA (codon adaptation tool JCAT)^2^. The sense and antisense analogous to the primary DNA sequence were synthesized (116 bp). The hybrid peptide FowlTα1 was expressed from the N-terminus by cloning it into the expression vector pPICZαA. This process involved the insertion of the peptide downstream of the in-frame α-factor secretion signal in the vector, using the restriction sites *EcoR I* and *Not I*. The stop codon (TGA) was positioned with a 6 × histidine tag at C-terminal to ease purification. PCR technique was used to acquire the full-length amplicon of the hybrid peptide (Fowl-Tα1) using the following primers and conditions: (P1; F. 5′ GAATTCTTGGTGATTAGAACG 3′; P2; R. 3′ GCGGCCGCAAAAGTGACCAG 5′), initial denaturation step at 94°C for 5 min, followed by 35 cycles of denaturation at 94°C for 40 s; annealing 55°C for 50 s, extension at 72°C for 50 s and a final elongation step at 72°C for 10 min/20s. The PCR product was digested with *EcoR I* and *Not I* enzymes and ligated into the *EcoR I/Not I*-digested pPICZα-A. Finally, the constructed recombinant peptide expression vector pPICZαA-FowlTα1 was transmuted into competent cells *E. coli* DH5α for cloning and confirmed by sequencing.

#### Collection of positive colonies and transformation into yeast

The expression plasmid pPICZα-AFowlTα1 was linearized with *Sac I* enzyme following electroporation manufacturer’s instructions. The blank pPICZα-A expression vector was inserted into *P. pastoris* SMD 1168 cells that served as a negative control. Afterwards, the transformed cells were grown on a medium of 1% yeast extract, 2% peptone, 2% dextrose, 1 M sorbitol (YPDS), 2% agar and 100 μg/mL Zeocin. Additionally, zeocin resistant cells were screened for positive insertion of the coding DNA for the hybrid peptide FowlTα1 utilizing PCR and DNA sequencing (Sanger Sequencing, BGI Genomics, Beijing, China).

#### Recombinant hybrid peptide FowlTα1 expression into *P. pastoris*

Recombinant peptide was expressed under optimal condition (0.5% methanol v/v, pH 5.5, and temperature 28°C) in Buffered Methanol-Complex medium (BMMY). The positive yeast cells were cultured for 22 h in a shaking flask inclosing 1% yeast extract, 2% peptone, 100 mM potassium phosphate buffer, pH 5.0, 1.34% YNB, 4 × 10^–5^% biotin and 1% glycerol (BMGY) 50 ml to OD_600_ = 4.0. Afterwards, the cells were harvested by centrifugation (2,000 × *g*) for 12 min at 25°C. Subsequently, the cells were resuspended in BMMY medium (1% yeast extract, 2% peptone, 100 mM potassium phosphate buffer, pH 5.0, 1.34% YNB, 4 × 10^–5^% biotin, and 0.5% methanol) to promote the expression of the recombinant hybrid peptide. Of the expression medium, 50 μL were analyzed by Tricine-sodium dodecyl sulfate-polyacrylamide gel electrophoresis (Tricine SDS-PAGE) after 120 h methanol induction. The concentration of expressed peptide was determined via Bradford method utilizing bovine serum albumin (BSA) as standard (Bradford protein assay kit, Sangon Biotech, Shanghai, China).

#### Purification of recombinant hybrid peptide FowlTα1

The crude hybrid peptide FowlTα1 was dissolved in a solvent mixture of water and acetonitrile (ACN), with the addition of 0.1% trifluoroacetic acid (TFA). This solution was then introduced into a RP-HPLC system equipped with a C18 column. Separation was achieved using a gradient elution involving water (containing 0.1% TFA) and acetonitrile (also containing 0.1% TFA), and the process was monitored at a wavelength of 214 nm. The hybrid peptide fractions were collected based on retention time and analyzed for purity using analytical HPLC. Fractions with purity > 95% were pooled, lyophilized, and stored for subsequent experiments (Parachin et al., 2012). The eluted fractions were analyzed using Tricine-SDS-PAGE, blue staining and Bandscan 5.0 software. After purification, the hybrid peptide was diluted in milli-Q water and filtered through a 0.22 μM filter. It was then prepared for ESI-MS/MS.

#### Antimicrobial effectiveness of hybrid FowlTα1 peptide on gram-negative bacteria

The antimicrobial activity of FowlTα1 was tested against *E. coli* C 84002 using agar well diffusion. The indicator strain was diluted and spread onto Mueller-Hinton broth (MHB) plates. Subsequently, 6 mm wells were punched out from the agar using a steel cylinder and 100 μL of purified recombinant FowlTα1 or media without peptide and D-PBS were added to each well. Ampicillin (100 U, 10 mg/L) served as a positive (inhibitory) control. The inhibition zone was measured after overnight incubation at 37°C.

#### Lipopolysaccharide (LPS) neutralization of hybrid peptide FowlTα1

To evaluate the neutralization of LPS activity of the parental (Fowl) and hybrid (FowlTα1) peptides, achromogenic Limulus amebocyte lysate (LAL) assay was performed. LPS (1 EU/mL) and dissimilar concentrations of peptides (20–50 μg/mL) were incubated at 37°C. An equal volume (50 μL) of LAL reagent was mixed with the sample and incubated at 37°C for 10 min. After adding 100 μL of a chromogenic substrate solution, the solution turned yellow. The reaction was stopped by adding HCL and absorption was measured at 545 nm (Wang et al., 2019).

#### Hemolytic activity

The hemolytic activity of the hybrid peptide FowlTα1 was measured using primary chicken red blood cells (RBCs). The fresh chicken red blood cells (RBCs) were washed twice with 1x PBS and then diluted to a hematocrit of 10%. The cells were treated with hybrid peptide and parent peptides at various concentrations (20–50 μg/ml) for 1 h at 37°C. The absorbance of the culture supernatant was analyzed at 414 nm. Triton X-100 and PBS were used as the positive and negative controls, respectively.

#### Cell culture

Chicken HD11 macrophages were obtained from China Agricultural University and grown in Dulbecco’s modified Eagle’s medium (DMEM) supplemented with 1% streptomycin, 10% fetal bovine serum and 1% penicillin. Cells were propagated by subculturing in a humidified cell incubator at 37°C with 5% CO_2_. Before inoculation, cells were cultured for 48 h to reach approximately cell density of 80%. After inoculation, the cells were maintained in the incubator for 24 h to allow proper infection and treatment.

#### Lactate dehydrogenase activity (LDH) assay

The LDH assay was used to evaluate the cytotoxic effect of a hybrid peptide on HD11 chicken macrophages. LPS-induced cells (1 μg/mL, 1 × 10^5^ cells/mL) and controls (1 × 10^5^ cells/mL, no LPS) were exposed to parental and hybrid peptides (20–50 μg/mL) and incubated for 24 h with slight modifications. The supernatants were collected, and the cytotoxic levels were measured according to the kit instructions (Dojingdo Laboratories, Kumamoto, Japan).

#### Inhibition of nitric oxide (NO) production in LPS-stimulated chicken HD11 macrophages

The chicken HD11 cells were incubated with LPS only (1 μg/ mL) and LPS plus the different concentrations of parental and hybrid peptides (20–50 μg/mL). NO production was quantified from the collected supernatant. A 100 μL aliquot of the culture medium was combined with an equal volume of Griess reagent (1% sulfanilamide in 5% phosphoric acid and 0.1% naphthyl ethylenediamine dihydrochloride) and incubated for 15 min. The concentration of NO was measured spectrophotometrically at 550 nm using an enzyme-linked immunosorbent assay (ELISA) reader.

#### Assessing the levels of pro-inflammatory cytokines in chicken HD11 macrophages induced by LPS

Following the addition of LPS (1 μg/mL) to HD11 cells (5 × 10^5^ cells/well) in the presence or absence of Fowl and hybrid FowlTα1 peptide (20–50 μg/mL), the expression levels of pro-inflammatory cytokines, including TNF-α, IL-1β, and IL-6, were evaluated using a commercial ELISA kit (Cytokines, Cloud-Clone Corp, Houston, USA). The cytokine levels were quantified by measuring absorbance at 450 nm.

### Statistical analyses

All the data were presented as mean ± S.D. For statistical analysis, one-way analysis of variance (ANOVA) and Duncan’s multiple range tests were used and carried out with SPSS 19.0 (SPSS Inc., Chicago, IL, USA). Differences with *P* < 0.05 were considered statistically significant.

## Results

### Structure analysis of hybrid peptide FowlTα1

**FIGURE 1:**
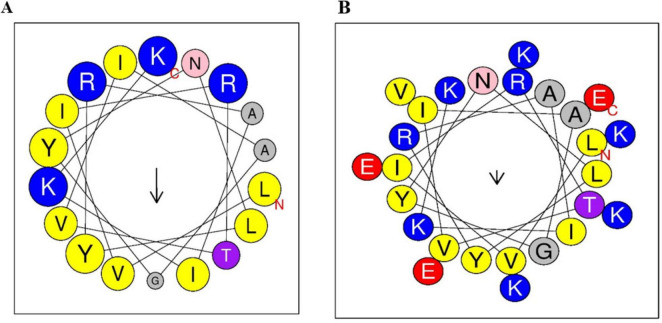
Helical wheel projections of antimicrobial peptides. **(A)** Fowlicidins (Fowl) peptide shows a typical amphipathic helical structure with hydrophobic and hydrophilic residues segregated. **(B)** Hybrid FowlTα1 peptide displays enhanced amphipathic character. The hydrophobic residues are highlighted in yellow, positively charged residues are in blue, and neutral amino acids are represented in gray or pink.

The Fowl peptide is composed of hydrophobic residues shown in yellow, positively charged in blue, and neutral amino acids depicted in gray/pink. The helical wheel plot illustrates an ideal amphipathic structure, with hydrophobic residues predominantly localized on one side of the helix, while positively charged residues such as Lysine (K) and Arginine (R) are concentrated on the opposite side (Figure 1A). Furthermore, hybridizing with Tα1 (KEKKEVE) further enhanced the amphipathic nature of the peptide, as illustrated by the helical wheel plot. The increased presence of positively charged residues, such as lysine (K), arginine (R), and glutamate (E), enhances the segregation of hydrophobic and hydrophilic faces, which is anticipated to improve the antimicrobial and immunomodulatory efficacy of the peptide (Figure 1B). The physicochemical properties of both the parental Fowl peptide and the hybrid FowlTα1 peptide are detailed in Table 1, highlighting key differences in charge, hydrophobicity, amphipathicity, and overall molecular composition.

**TABLE 1:** Summary of the physicochemical properties of Fowl, Tα1 and hybrid FowlTα1 peptides, including sequence length, molecular weight (MW), isoelectric point (pI), net charge, hydrophobicity, and toxicity prediction.

Peptides	Length	MW	pI	Net charge	Hydrophobicity	Prediction
Fowl	19	2,218	10.99	+5	+14.97 Kcal* mol^–1^	Non-toxin
Tα1	7	888	6.77	+0	+26.73 Kcal* mol^–1^	Non-toxin
FowlTα1	26	3,088	10.46	+6	+33.80 Kcal* mol^–1^	Non-toxin

### Construction and expression of pPICZαA-FowlTα1

A hybrid peptide gene (GAATTCTTGGTGATTAGAACGGT CATTGCGGGTTACAACTTGTACAGAGCAATAAAGAAAAAA AAGGAGAAAAAGGAAGTTGAATAGCATCATCATCATCATC ATTGAGCGGCCGC) was amplified using PCR. The resulting 116 bp DNA fragment encodes a C-terminal 6 × His tag. The tag was flanked by *EcoR I* and *Not I* restriction enzyme sites at its 5′ and 3′ ends, respectively. Subsequently, the modified fragment was cloned into the pUC57 vector. The fragment was subjected to double-digestion using the specified restriction enzymes. It was then frame cloned to attach to the 3′ end of the α-factor secretion signal, downstream of the AOX1 promoter in the expression plasmid pPICZαA. This resulted in the creation of a recombinant vector named pPICZαA-FowlTα1. The insertion was confirmed by restriction enzyme digestion analysis and sequencing. The process of constructing pPICZαA-FowlTα1 is illustrated in Supplementary Figure 1. The plasmid vector pPICZαA-FowlTα1 was treated with *Sac I* enzyme and the resulting linear fragment was transformed into the competent cells of *P. pastoris* SMD 1168 by electroporation. Twenty colonies that showed resistance to zeocin (100 μg/mL) were selected and confirmed by PCR using specific primers for FowlTα1 and pPICZAαA. Our results confirmed that the target pPICZαA-FowlTα1 sequence was successfully integrated into the host cells. The colonies that had undergone positive transformation were induced using 1% pure methanol (v/v) 120 h after optimizing the methanol concentration. After cells were induced by methanol, the culture supernatant was collected and analyzed using Tricine-SDS-PAGE and blue staining. As anticipated, the recombinant hybrid FowlTα1 peptide with a molecular weight of 3.1 kDa was observed (Supplementary Figure 2).

### Purification, RP-HPLC, and mass spectrometry analysis of hybrid FowlTα1 peptide

**FIGURE 2:**
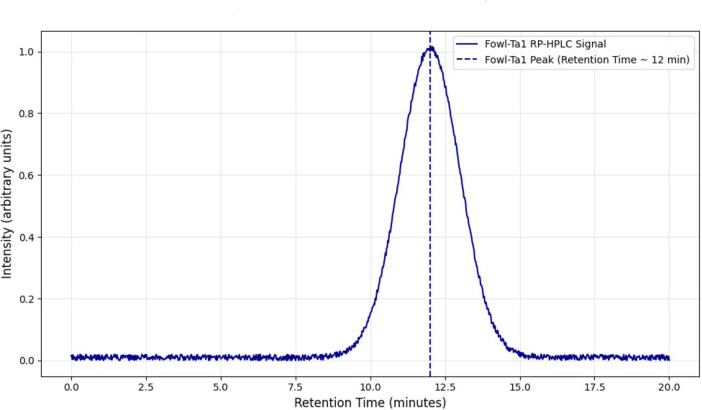
Reverse-phase high-performance liquid chromatography (RP-HPLC) of the purified hybrid peptide using a C18 column. The retention time of the hybrid peptide was observed at 12.5 min, indicated by the prominent peak. The analysis confirms the successful purification of the peptide with high purity.

**FIGURE 3:**
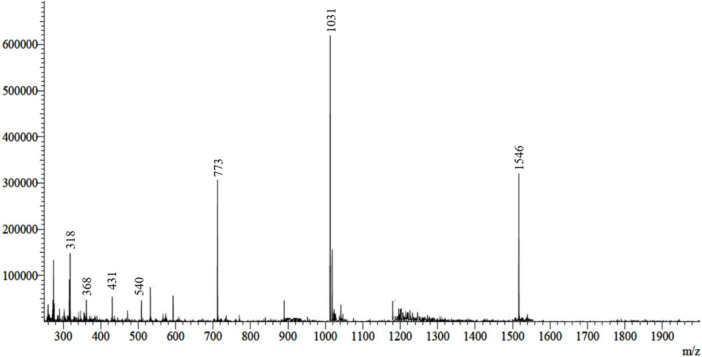
ESI-MS analysis of the recombinant purified hybrid peptide. The spectrum displays the molecular ion peaks corresponding to the expected mass, confirming the successful expression and purification of the hybrid peptide.

The FowlTα1 peptide was obtained from the culture medium after centrifugation and subjected to purification using the RP-HPLC method. The crude peptide was eluted with a retention time of approximately 12 min (Figure 2), resulting in a highly purified product with a final yield of 7.2 mg. The purified peptide was analyzed using SDS-PAGE, where a single band corresponding to the estimated size of 3.1 kDa was observed, confirming the successful expression and purification of the hybrid peptide as shown in Supplementary Figure 3. Mass spectrometry of the purified FowlTα1 displayed a single non-dispersed signal (Figure 3). The average mass of the molecular ion was [M+4H+]^4+^ 773 Da, [M+3H+]^3+^ 1031 Da, [M+2H+]^2+^ 1546 Da and successful removal of the N-terminus from the recombinant FowlTα1 peptide was confirmed by the resulting molecular mass of 3090 Da. Our results revealed that the hybrid recombinant peptide was removed from the C-terminus successfully.

### Recombinant hybrid peptide FowlTα1 neutralize endotoxin

**FIGURE 4:**
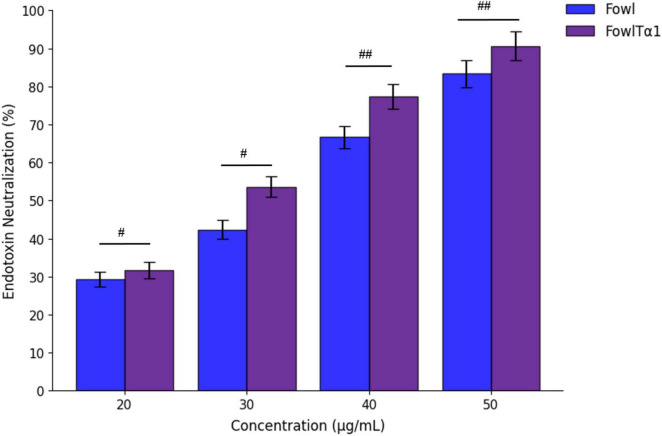
Endotoxin neutralization activity of the parental peptide Fowl (used as a control) and the hybrid peptide FowlTα1. Mean values from three independent trials are displayed (*n* = 3) and presented as mean ± standard deviation (SD). Statistical analysis was performed using one-way ANOVA followed by Duncan’s multiple range test. Statistical significance was indicated as *^#^p* < 0.05 and *^##^p* < 0.01, comparing Fowl vs. FowlTα1.

The FowlTα1 peptide has a net charge of +6. After physical characterization, we predicted that it would bind and neutralize the endotoxin. We tested the neutralization capability of the parental and hybrid peptide in vitro using the LAL test, which is a highly sensitive indicator of the presence of free non-neutralized endotoxin. Our findings indicate that Fowl at concentrations of 40 and 50 μg/mL can effectively neutralize endotoxin, with a neutralization rate of 66.65 ± 2.51% and 77.33 ± 2.78 %, respectively. That dose-dependent neutralization effect was even higher for FowlTα1, with a rate of 83.33 ± 1.56% and 90.66 ± 1.20% at concentrations of 40 and 50 μg/ml, respectively. Thus, the hybrid peptide exhibited a significant increase in the neutralization of endotoxin as compared to the parental peptide (Figure 4).

### Cytotoxicity and hemolytic activity

**FIGURE 5:**
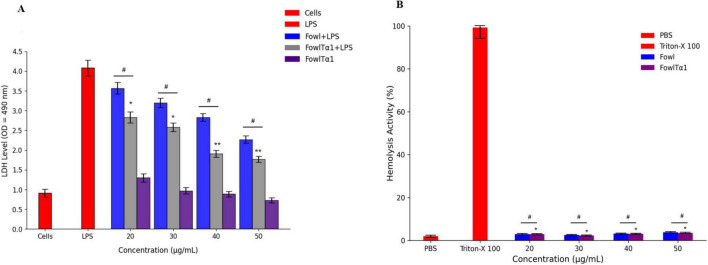
**(A)** Cytotoxic effect in endotoxin-exposed chicken HD11 macrophages. Experiments were conducted in a DMEM culture medium for chicken HD11 cells. Data are presented as mean ± SD from three independent trials. Statistical significance was determined using one-way ANOVA followed by Duncan’s multiple range test (*p < 0.05; **p < 0.01 vs. LPS), while ^#^*p* < 0.05 indicates a significant difference between the Fowl and FowlTα1 peptides. **(B)** Hemolysis evaluation of chicken RBCs. Data represent the mean values from three independent trials and are expressed as a percentage of hemolysis ± SD. Statistical significance was revealed using one-way ANOVA followed by Duncan’s multiple range test (*p < 0.05 vs. Triton X-100, the positive control), while *^#^p* < 0.05 indicates a significant difference between the hybrid (FowlTα1) and parental (Fowl) peptides.

To determine whether the recombinant hybrid FowlTα1 peptide can be developed as a potential anti-inflammatory agent, it is important to assess its interaction with LPS in the presence of cells. Therefore, we conducted a study to examine potential mitigation of LPS-induced cell damage such as cytotoxicity in HD11 macrophages and lysis of chicken erythrocytes. Our results exhibited that, as expected endotoxin exposure in the absence of the peptide revealed a significantly higher level of LDH release at 24 h, compared to the control group. However, when cells were exposed to endotoxin in the presence of FowlTα1 peptide, that LDH release was significantly lowered, compared to endoxin-only exposure. This outcome confirms that LPS severely damaged the chicken HD11 macrophages. However, different concentrations of the hybrid FowlTα1 peptide were able to neutralize the LPS and reduce the LDH level to 1.9 ± 0.072 at 40 μg/mL and 1.7 ± 0.040 at 50 μg/mL, respectively (Figure 5A). Additionally, the hybrid peptide was found to be more effective than the parental peptide in reducing LPS-induced cytotoxicity. When it comes to hemolysis, cells treated with the hybrid peptide showed a significant decrease in hemolysis (*p* < 0.01) compared to the Triton X 100 positive (hemolytic) control, with < 1% hemolysis observed for the hybrid peptide (Figure 5B). An LDH-assay using the hybrid peptide only (no LPS added) did not deviate significantly from the negative control. These findings suggest that the hybrid FowlTα1 peptide does not have significant cytotoxic or hemolytic properties in concentrations that did effectively neutralize endotoxin.

### Effect against gram-negative bacteria

**FIGURE 6:**
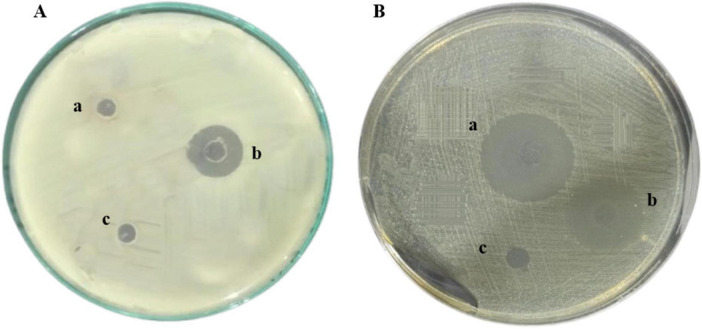
Antimicrobial Activity of Recombinant FowlTα1 against *E. coli* C84002. **(A)** The treatments included (a, c) a negative control using sodium phosphate buffer (PBS); (b) recombinant hybrid FowlTα1 peptide at a concentration of 5 mg/L. **(B)** (a) Hybrid FowlTα1 peptide at a concentration of 5 mg/L; (b) 10 mg of ampicillin; (c) control PBS. The Fowl-Tα1peptide displayed a larger inhibition zone compared to ampicillin and the control.

The antimicrobial effectiveness of the hybrid peptide FowlTα1 was evaluated using the agar well diffusion method against common Gram-negative bacterial pathogens. The purified FowlTα1 peptide exhibited significant inhibitory activity against *E. coli* C84002. Compared to ampicillin a larger inhibition zone was noted with the hybrid peptide (18 mm vs. 22 mm; (Figures 6A, B). The findings demonstrate that the recombinant hybrid FowlTα1 effectively inhibits the growth of Gram-negative bacteria, highlighting its potential as a potent antimicrobial agent.

### FowlTα1 down regulates LPS-stimulated inflammatory response in chicken HD11 macrophages

**FIGURE 7:**
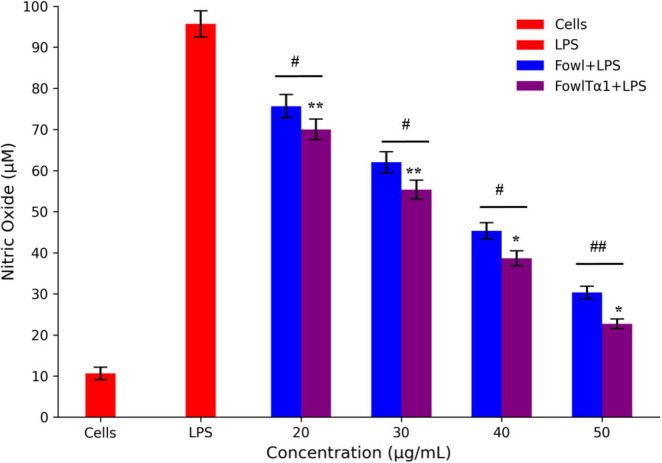
Nitric oxide secretion levels in HD11 avian macrophages challenged with LPS. Data are presented as mean ± SD from three independent trials. Statistical significance was analyzed using one-way ANOVA followed by Duncan’s multiple range test, comparing all groups to the LPS control group (*p < 0.05; **p < 0.01). Additionally, significant differences between the Fowl and FowlTα1 groups are indicated as *^#^p* < 0.05; ^##^*p* < 0.01.

**FIGURE 8:**
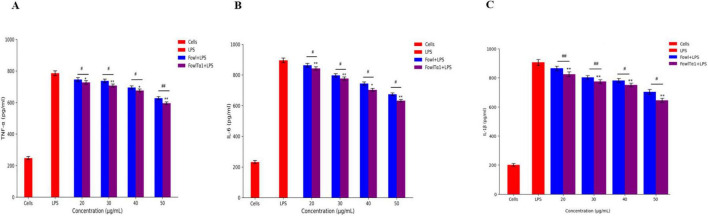
Hybrid peptide FowlTα1 and parental peptide Fowl modulate endotoxin-induced inflammatory responses in chicken HD11 macrophages. **(A)** TNF-α, **(B)** IL-6, and **(C)** IL-1β inhibition were measured after 24 h of incubation in endotoxin-stimulated cells treated with different doses of Fowl and FowlTα1 peptides. Data are presented as mean ± SD from three independent trials. Statistical significance was analyzed using one-way ANOVA followed by Duncan’s multiple range test (*p < 0.05; **p < 0.01 vs. endotoxin control). Additionally, significant differences between the Fowl and FowlTα1 groups are indicated as ^#^*p* < 0.05; ^##^*p* < 0.01.

The ability of FowlTα1 to counteract the effects of LPS leads us to believe that it might also be able to reduce the inflammatory response triggered by LPS. To investigate this possibility, we examined the impact of a hybrid FowlTα1 peptide on the production of nitric oxide (NO) and pro-inflammatory cytokines such as TNF-α, IL-6, and IL-1β in chicken HD11 macrophages that had been exposed to LPS. According to our study, the level of NO in chicken HD11 macrophage cells significantly increased after treatment with LPS, as compared to the control group 95.2 μM vs 10.66 μM. However, after the administration of FowlTα1at a concentration of 40 and 50 μg/mL the NO level was abridged to 38.23 μM and 22.75 μM, as illustrated in Figure 7. Compatibly, we analyzed the levels of TNF-α, IL-6, and IL-1β in chicken macrophages using ELISA. Our findings revealed that FowlTα1 was able to significantly reduce the secretion of these cytokines in the cells compared to cells infected with LPS only. Specifically, treatment with FowlTα1 at concentrations of 40 and 50 μg/mL reduced the concentration of TNF-α by 676 pg/ml and 595 pg/ml, respectively, IL-6 by 703 pg/ml and 632 pg/ml, and IL-1β by 751 pg/ml and 648 pg/ml, respectively (Figures 8A–C). These results suggest that FowlTα1 has potent anti-inflammatory properties, and is even more effective than the parental peptide Fowl (Hu et al., 2017; Zhu et al., 2024).

## Discussion

Antimicrobial peptides play a critical role in innate immunity by targeting a wide range of pathogens, including bacteria, fungi, parasites, and viruses (Kang et al., 2017; Mohammed et al., 2017; Kang et al., 2019; Lei et al., 2019). With the increasing challenge of antibiotic resistance (Chung and Khanum, 2017; Hiltunen et al., 2017), AMPs offer a viable alternative due to their ability to disrupt microbial membranes and intracellular structures (Chung and Khanum, 2017; Mohammed et al., 2017; Mwangi et al., 2019). However, optimizing AMPs for therapeutic applications requires balancing efficacy, stability, and biocompatibility (Nikaido and Vaara, 1985; Liu et al., 2018; Jenner et al., 2019). In the present study, we successfully developed and expressed a novel hybrid peptide FowlTα1 in *P. pastoris*, demonstrating its potential as an effective antimicrobial and anti-inflammatory agent.

The hybridization of Fowl and Thymosin α1 was strategically designed to enhance amphipathic properties, which is crucial for effective interaction with bacterial membranes and endotoxins (Nikaido and Vaara, 1985; Liu et al., 2018). Our findings align with previous studies that showed increased efficacy in hybrid peptides compared to their parental counterparts, such as the hybrid peptide LL-37Tα1, which demonstrated enhanced immunomodulatory effects (Ahmad et al., 2019). Similarly, CATH-2TP5 and DEFB-TP5 exhibited superior anti-inflammatory properties in comparison to their original peptides (Ahmad et al., 2020a; Ahmad et al., 2020b). In the current study, the helical wheel projection of FowlTα1 confirmed an optimal structural arrangement that facilitates membrane disruption and endotoxin neutralization. This dual mechanism not only improves antimicrobial activity but also strengthens host immune regulation, a feature lacking in conventional antibiotics (Xiao et al., 2009; Liu et al., 2025).

The recombinant production of FowlTα1 in *P. pastoris* demonstrated advantages over bacterial expression systems, including higher yield, reduced cytotoxicity and improved post-translational modifications (Ahmad et al., 2014; Ahmad et al., 2019). Notably, the expression levels of FowlTα1 (110 mg/L) were superior to the previously reported hybrid peptides expressed in bacterial systems, such as ceropin AD (80 mg/L) and CA-MA (90 mg/L) (Ahmad et al., 2014; Lee et al., 2016). This result suggests that yeast-based expression systems could serve as a sustainable approach for large-scale AMP production, addressing economic and scalability concerns in biopharmaceutical applications (Ahmad et al., 2014).

In the present study, FowlTα1 exhibited significant antimicrobial activity against *E. coli* C84002, surpassing conventional antibiotics in inhibition zone assays (Xiao et al., 2009). Compared to DEFB-TP5, which displayed moderate antibacterial activity, FowlTα1 exhibited a larger inhibition zone, indicating enhanced potency (Ahmad et al., 2020b). Its ability to neutralize LPS was demonstrated through the LAL assay, where it showed a higher neutralization rate compared to the parental peptide (Lee et al., 2016). This suggests that the hybrid peptide not only eradicates bacterial infections but also mitigates inflammation induced by endotoxins, a critical factor in sepsis and other inflammatory conditions (Lee et al., 2016). Additionally, cytotoxicity and hemolysis assays confirmed that FowlTα1 is biocompatible, with minimal adverse effects on host cells (Zhang et al., 2015; Ahmad et al., 2020a). This is a key advantage over synthetic antibiotics, which often cause cytotoxicity and disrupt normal microbiota (Zhu et al., 2024). Similar to previous studies on SPHF1, which showed low cytotoxicity and effective LPS neutralization, FowlTα1 exhibited comparable safety and enhanced efficacy (Ahn et al., 2012; Liu et al., 2025). Moreover, in LPS-stimulated avian macrophages, FowlTα1 significantly reduced nitric oxide and pro-inflammatory cytokine levels (TNF-α, IL-6, and IL-1β), highlighting its immunomodulatory potential (Du et al., 2025; Lin et al., 2025). In the present study, our findings indicate that FowlTα1 not only neutralizes pathogens but also prevents excessive immune responses, reducing the risk of tissue damage and inflammatory diseases (Ahn et al., 2012). Compared to previously reported hybrid peptides, FowlTα1 demonstrates superior antimicrobial potency, higher yield, and enhanced LPS neutralization, making it a strong candidate for future therapeutic applications (Li et al., 2024; Lin et al., 2025; Liu et al., 2025). The combination of antimicrobial, endotoxin-neutralizing, and immunomodulatory properties positions FowlTα1 as a next-generation therapeutic agent.

## Conclusion

We have developed a successful method for expressing the hybrid FowlTα1 peptide in *P. pastoris* using the expression plasmid PpICZαA. FowlTα1 is highly effective in neutralizing LPS without displaying cytotoxic or hemolytic activity. Moreover, the novel FowlTα1 peptide has demonstrated antimicrobial, immunomodulatory and anti-inflammatory properties by inhibiting the release of cytokines such as NO, TNF-α, IL-6, and IL-1β, LPS-induced damages in HD11 macrophages and chicken erythrocytes. In addition to these findings, the recombinant production of FowlTα1 offers a sustainable and eco-friendly alternative to traditional antibiotic synthesis, addressing concerns about antimicrobial resistance and environmental impact. Its dual functionality of neutralizing endotoxins while exhibiting antimicrobial activity positions it as a next-generation therapeutic agent for animal health applications.

## Data Availability

The datasets presented in this study can be found in online repositories. The names of the repository/repositories and accession number(s) can be found in the article/Supplementary material.

## Footnotes

1
https://web.expasy.org/protparam/

2
http://www.jcat.de/Start.jsp
