# Supplementary material for: Synergistic effects of recombinant expressed Fowlicidin and Thymosin α1 hybrid peptides in modulating inflammation and infection in avian macrophages
Source: Front Microbiol. 2025 Apr 16;16:1568451. doi: 10.3389/fmicb.2025.1568451 (PMC12042281; doi:10.3389/fmicb.2025.1568451)
Supplement: Supplementary file 1 [file Data_Sheet_1.pdf]

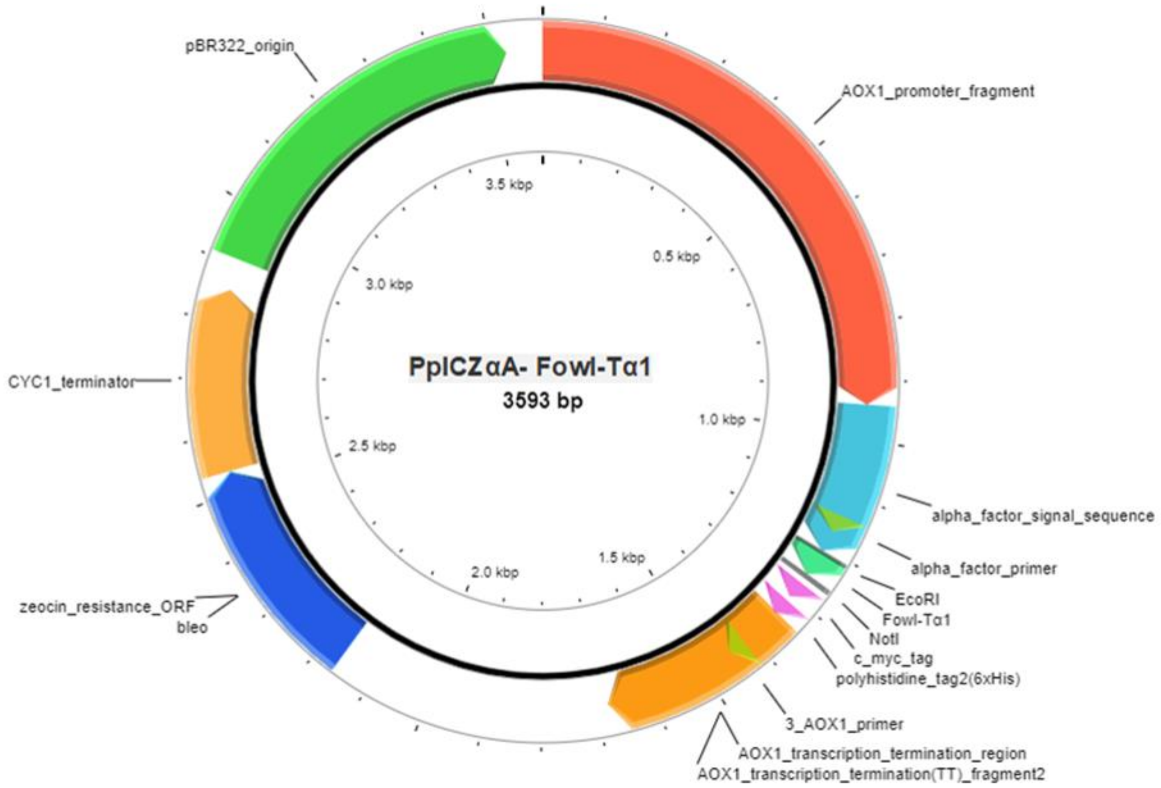

Supplementary Figure 1. Construction of recombinant plasmid pPICZαA-Fowl-Tα1.

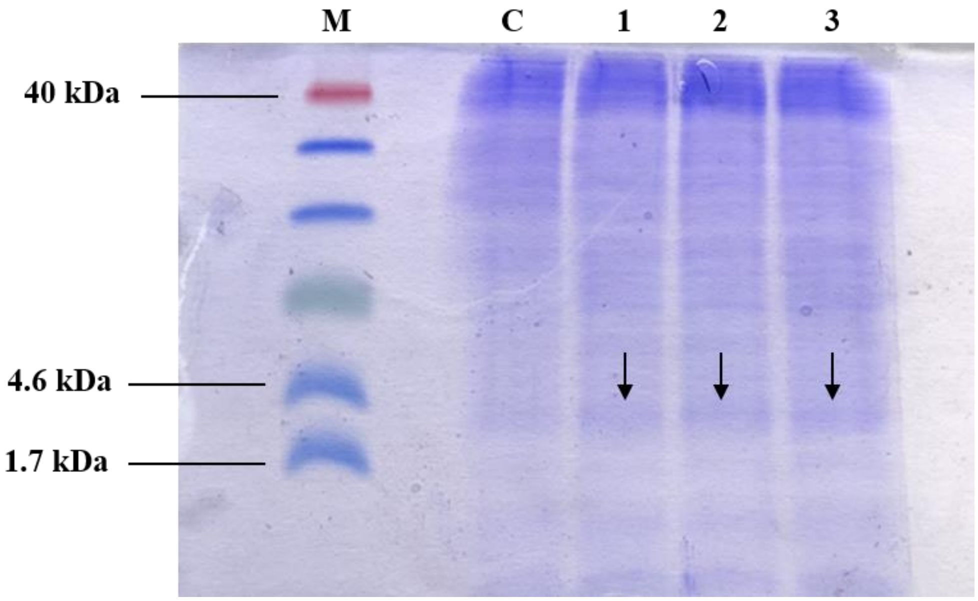

Supplementary Figure 2: Analysis of Recombinant Hybrid Peptide: Tricine-SDS-PAGE was performed on cell cultures from P. pastoris expressing the secreted Fowl-Tα1 peptide. Lane M contains molecular weight (MW) markers ranging from 1.7 kDa to 40 kDa. Lane C serves as the control, comprising the supernatant from a PpICZαA strain without the hybrid peptide. Lanes 1 to 3 display the results after 120 hours of methanol induction of the hybrid peptide, with an arrow indicating the molecular weight of the hybrid peptide at 3.1 kDa.

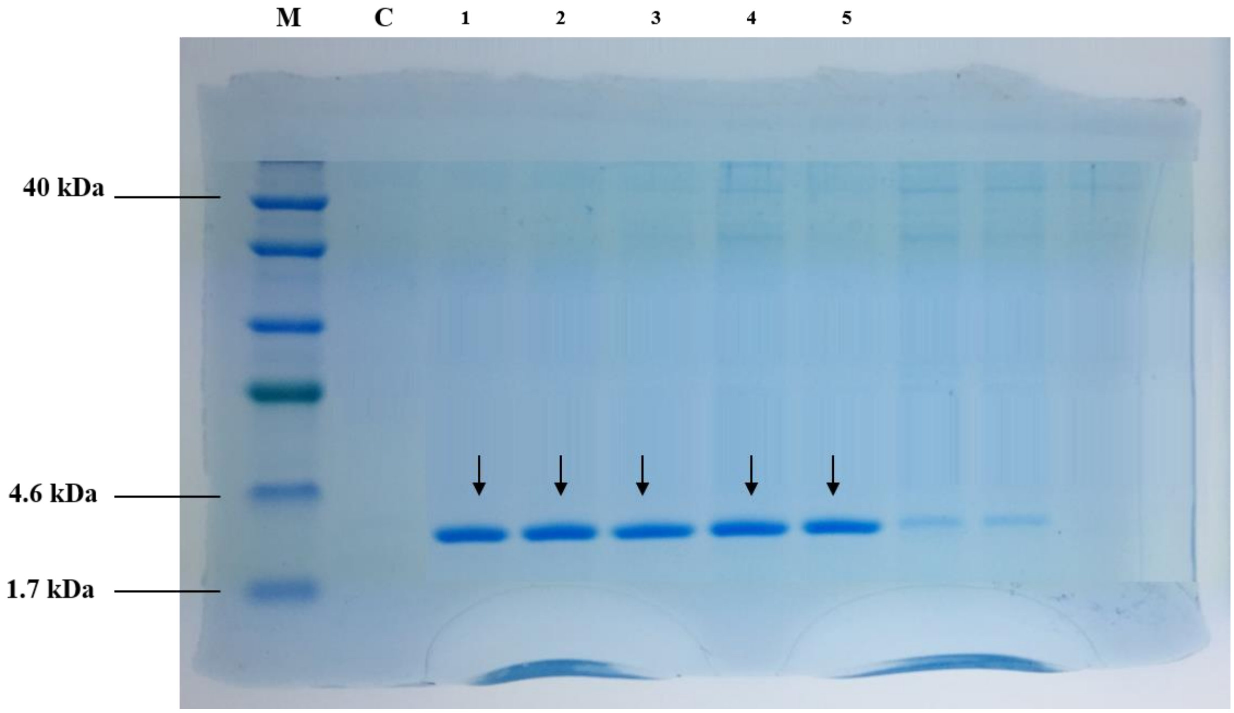

Supplementary Figure 3. Recombinant purified hybrid peptide Fowl-Tα1 was analyzed using Tricine-SDS-PAGE. Lane M shows the molecular weight (MW) markers, ranging from 1.7 kDa to 40 kDa. Lane C represents the control, which is the supernatant from SMD 1168 /PpICZαA without the hybrid peptide strain. Lanes 1 to 5 contain the purified expression of the hybrid peptide, with an arrow indicating the size of the hybrid peptide at 3.1 kDa.
